# Cutaneous Metastases as Initial Presentation of Lung Carcinoma

**DOI:** 10.7759/cureus.15344

**Published:** 2021-05-31

**Authors:** Gaurav Sharma, Pramit Kumar, Hardik Veerwal, Parneet Singh, Sweety Gupta, Vandana Dhingra

**Affiliations:** 1 Department of Radiation Oncology, All India Institute of Medical Sciences, Rishikesh, Rishikesh, IND; 2 Department of Nuclear Medicine, All India Institute of Medical Sciences, Rishikesh, Rishikesh, IND

**Keywords:** carcinoma lung, cutaneous metastasis, palliation, bone scan, 99mtc-mdp

## Abstract

Breast cancer, in women, and lung cancer, in men, are the most common origins of cutaneous metastasis. Lung cancer can metastasize to any organ but mostly to the contralateral lung, liver, adrenal gland, bones, and brain. Over 1-12% of patients with lung cancer can develop skin metastasis. Non-small cell lung cancer includes 87% of lung cancer cases. Adenocarcinomas subtype accounts for approximately 40% of all lung cancers and is the most common histology in women. A woman’s lifetime risk of developing lung cancer is 1 in 16 women but lower than that of a man (1 in 13 men). The survival rates of women with lung cancer are usually higher than those of men. Herein, we report the case of a 66-year-old female who presented with painless multiple skin nodules over the chest back and axilla for three months. On evaluation, biopsy and immunohistochemistry were done from skin nodules suggestive of adenocarcinoma. CT thorax showed lung mass and was diagnosed as a case of metastatic adenocarcinoma, primary being from the lung. Our case demonstrated that skin metastasis could be the first sign of internal malignancy. Metastasis to the skin is often a preterminal event that heralds a poor prognosis.

## Introduction

Lung cancer is one of the malignancies with very high incidence and mortality. According to GLOBOCAN 2018, the incidence of lung cancer in the world is 11.6% and the mortality rate is 18.4%. In India, the incidence and mortality rate of the same is 6.45% and 8.82%, respectively. There is gender variability in metastasis to the skin from lung cancer. In men, skin metastasis arises mostly from malignancies of the lung (24%), colon (19%), melanoma (13%), and oral cavity (12%). In women, skin metastasis arise mostly from malignancies of the breast (69%), colon (9%), melanoma (5%), ovaries (4%), and lung (4%) [1]. Very rarely skin metastasis from lung cancer can be the initial presentation. This cutaneous presentation of lung cancer has a poor prognosis with an average survival of three to five months [2,3]. The most common sites of skin metastases from lung cancer are the chest, abdomen, head, and neck [4-6].

## Case presentation

**Figure 1:**
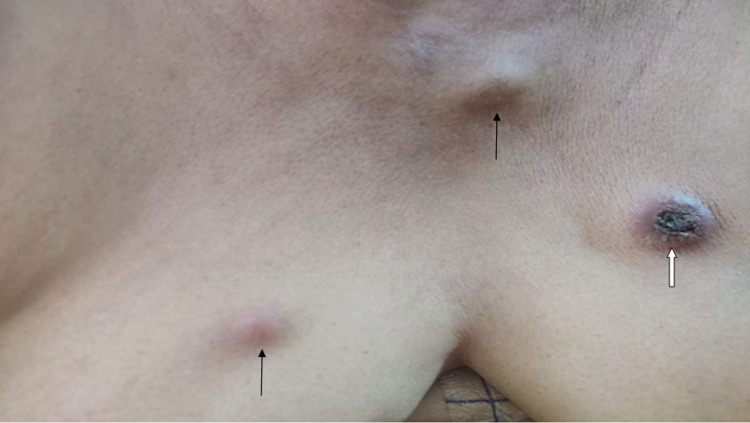
Multiple skin nodules (shown with arrows) present in the anterior chest wall. An ulcerated skin nodule is also shown (white solid arrow).

A 66-year-old female, a smoker for the last 30 years, was admitted to our department with chief complaints of breathlessness for two years and multiple skin nodules over the chest (Figure 1), back, axilla, head, and neck for three months. Her past medical history included chronic obstructive pulmonary disease for two years. Also, the patient has a history of weight loss, anorexia, and fatigue. Upon physical examination, multiple nodules present in the chest, back, a postauricular area which were firm, non-tender, skin-colored, and measured 1-4 cm in greatest dimensions. Bilateral level II neck nodes of size 1-1.5 cm were palpated which was non-tender, firm. Systemic examinations were done. The bilateral chest was normal. Bell’s palsy was noted on the right side of the face.

**Figure 2:**
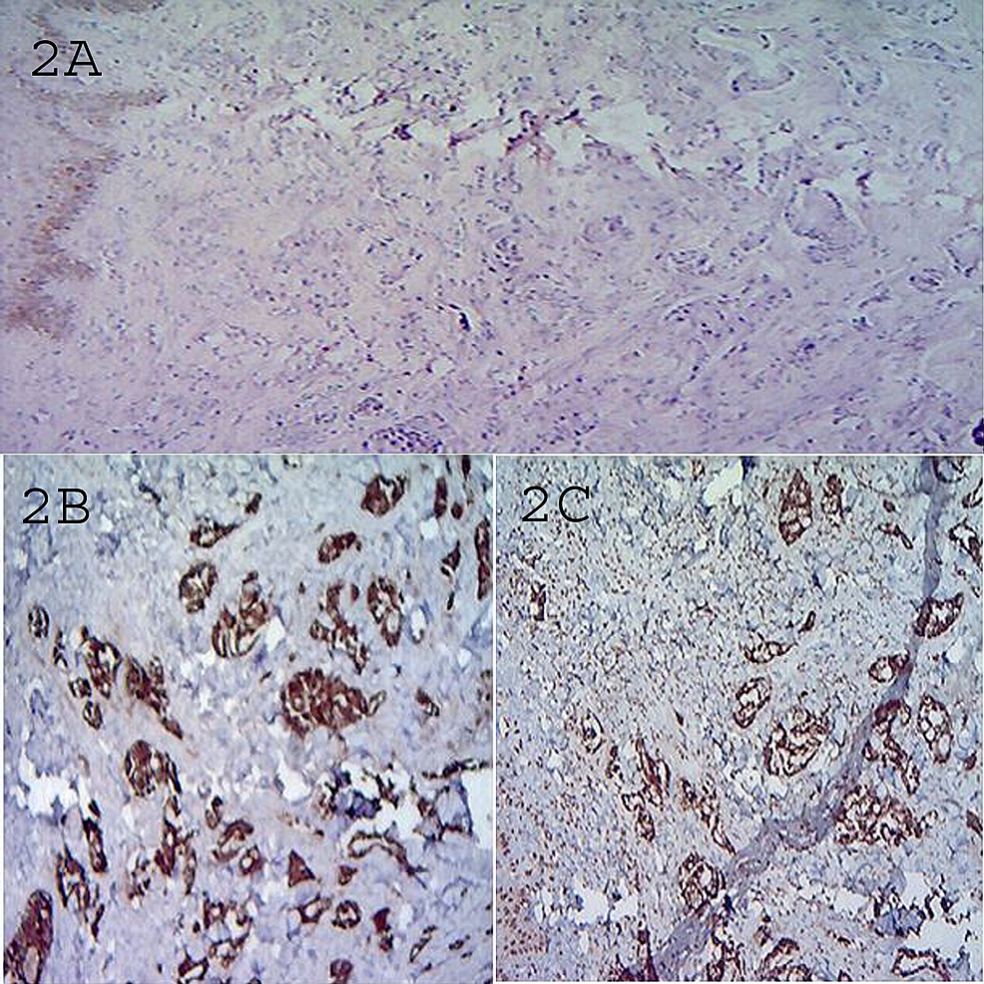
Hematoxylin and eosin staining (A) of microscopic sections of skin nodules confirms the diagnosis of adenocarcinoma lung. Immunohistochemical images revealed tumor cells positive for CK-7 (B) and TTF-1 (C). CK-7: cytokeratin-7, TTF-1: thyroid transcription factor-1.

Biopsy and immunohistochemistry were done from the skin nodules. The biopsy pattern consisted of cells arranged as acini and having increased nuclear size, coarse chromatin, conspicuous nucleoli with a moderate amount of cytoplasm (Figure 2A). In immunohistochemistry, tumor cells were positive for CK-7 (Figure 2B), TTF-1 (Figure 2C), and negative for CK-20. Histopathologically confirmed metastatic adenocarcinoma.

**Figure 3:**
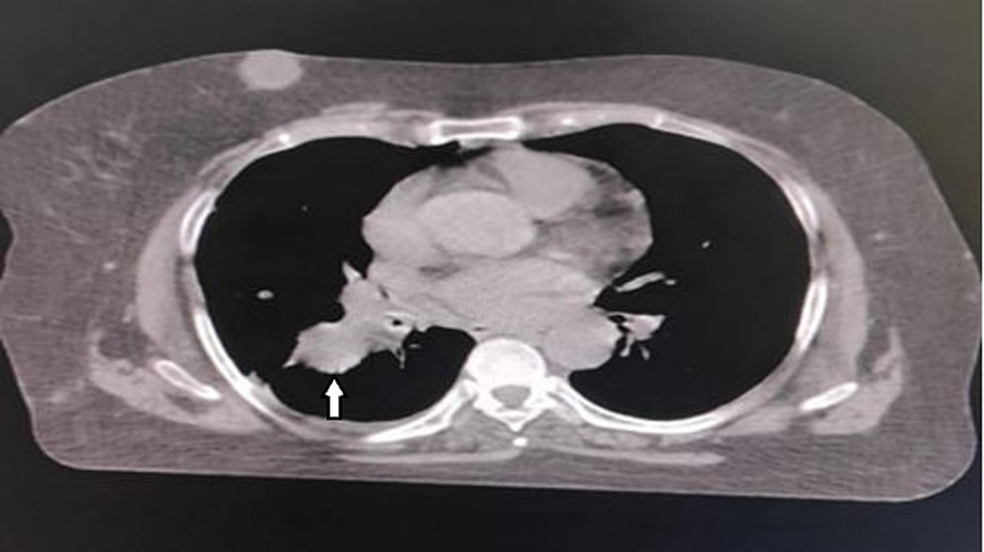
Transaxial CT image of thorax showing mass (marked with white arrow) in the perihilar superior segment of the right lower lobe

Subsequently, contrast-enhanced computer tomography (CECT) of the thorax was done which revealed a 4.7 × 2.5 × 3 cm^3^ mass lesion in the perihilar region of the right upper lobe (Figure 3), mediastinal lymphadenopathy, lytic lesion in D6 vertebra, multiple subcutaneous nodules in thoracic regions (largest 3.7 × 2.8 × 3 cm^3^).

CECT head was done suggestive of moderately heterogeneously enhancing lesion involving and causing destruction of right mastoid and eroding posterior part of right petrous temporal bones with the erosion of bony canal and mastoid segment of the facial nerve and stylomastoid foramen on the right side and compressing mastoid segment and eroding posterior wall of the right posterior semicircular canal.

**Figure 4:**
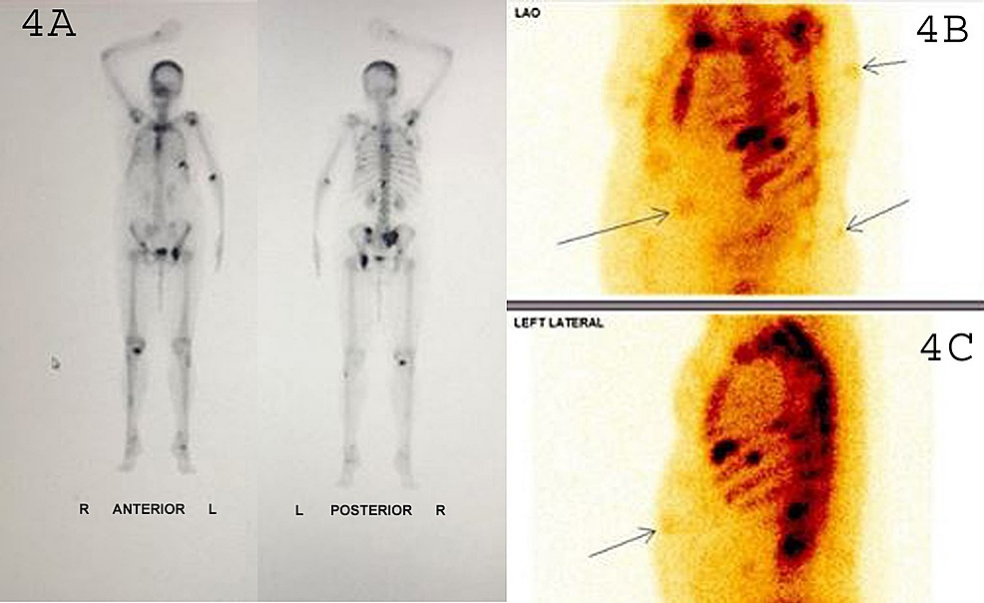
Planar bone scan images (A) in anterior and posterior views show multiple skeletal metastases. Left anterior oblique (B) and left lateral (C) view also reveals skeletal metastasis along with radiotracer uptake in multiple cutaneous nodules (shown with arrows).

Bone scan was done suggestive of multiple osteoblastic lesions (Figure 4A) in D6 vertebrae, pelvis, left humerus, right temporoparietal region, one lesion in rib, and interestingly multiple uptake sites corresponding to cutaneous nodules (shown with arrows in Figure 4B and 4C). A liver function test, kidney function test, and complete blood count were done. Raised serum alkaline phosphatase (ALP) level was consistent with the finding of skeletal metastases. Other laboratory parameters were within the normal limit.

The patient received palliative radiotherapy 20 Gy/5# to calvarial lesions and 8 Gy SFRT to pelvic bone lesions. Palliative chemotherapy was also planned for the patient.

## Discussion

In the majority of the patients, skin metastasis develops in the late course of the malignancy. Only in few cases, skin metastasis may occur as the initial presentation of malignancy [7]. In the present case, skin metastasis and primary cancer were diagnosed at the same time. Cutaneous metastasis occurs in 0.7-9% of all patients with cancer. The site of skin metastasis depends on the biology and location of the primary tumor and the sex. In a study, Brownstein et al. examined the distribution of skin metastasis in both sexes [1]. Skin metastasis is most commonly found in men among all primary malignancies. A meta-analysis in 2003 showed the incidence of cutaneous metastases in lung cancer was 3.4% in 89 patients among 2,597 subjects [8]. A retrospective study in 2012 indicated that 2.8% of 2,130 patients with advanced non-small-cell lung cancer (NSCLC) showed cutaneous metastases as an initial presentation [2]. Most of the skin metastasis occurs in regions that are close to the primary tumor. Lung cancers have a preference for metastasis to supra-diaphragmatic skin regions and colorectal cancers to infra diaphragmatic skin regions [9,10]. Cancer to the upper lobes of the lung also has a higher tendency to skin metastasis [11]. Possible lymphatic dissemination may be the reason for skin metastasis tending to near to primary tumor. Sites of presentation of skin nodules in the present case were supradiaphragmatic which was in par with the above studies. 

The presentation of skin metastasis is fast-growing solitary or multiple nodules with a diameter of 0.5-10 cm and are firm, mobile, and covered with normal skin or sometimes ulcerated also. In our case, the maximum size of the nodule was 4 cm in diameter and were firm, normal skin covering and at places hyperpigmentation also present.

Histology of lung cancer has also variability in skin metastasis. The most common histologic type of carcinoma of the lung that metastasizes to the skin is adenocarcinoma followed by squamous cell carcinoma, then small cell and large cell carcinoma [7]. The histology of our case is adenocarcinoma which has comparatively higher chances of skin metastasis according to the above study. But according to other studies, large cell carcinoma has the highest incidence of skin metastasis followed by adenocarcinoma [11].

In our patient, the bone scan revealed uptake of 99mTc-MDP at multiple sites corresponding to cutaneous nodules. Lung cancer metastasizing to the skin is a rare phenomenon with involvement in only 2.8-8.7% of cases [12]. On 99mTc-MDP bone scintigraphy, visualization of metastatic cutaneous nodules in adenocarcinoma lung is rarely reported.

Extra osseous uptake of 99mTc-MDP by a variety of primary malignancies including lung, breast, stomach, kidney, urinary bladder, oesophageal, colorectal cancers, etc., have been widely reported [13]. Plausible causes of uptake of 99mTc-MDP by the primary or secondary tumor may be described as metastatic calcification, dystrophic calcification, metabolic uptake, compartmental sequestration, or spurious artifacts. Mucinous adenocarcinoma tumors of the lung synthesize a glycoprotein that has biochemical similarity to ossifying cartilage [14]. The binding of Ca^2+^ salts to these glycoproteins is a possible explanation of metabolic uptake of 99mTc-MDP by cutaneous metastasis in this patient. Dystrophic calcification of the ischemic or necrosed metastatic tissue deposit in the skin [14] might also be a possible explanation for visualization of skin lesions on bone scan in this scan.

Patient with early presentation of skin metastasis and along with extracutaneous metastasis has a poor prognosis [15]. In our patient, along with skin metastasis, there were multiple bone metastasis also, which puts the patient into a very poor prognosis group. We have given palliative radiotherapy to bony lesions and planned for further treatment with chemotherapy.

## Conclusions

Skin metastasis is a rare presentation of lung cancer. But with the corroborative history of smoking, breathlessness and the supra-diaphragm lesions indicated the mild suspicion of malignancy even though other benign conditions were present. The prognosis of skin metastasis along with other distant metastasis is very poor even after any type of treatment.
